# Selective directed self-assembly of coexisting morphologies using block copolymer blends

**DOI:** 10.1038/ncomms12366

**Published:** 2016-08-02

**Authors:** A. Stein, G. Wright, K. G. Yager, G. S. Doerk, C. T. Black

**Affiliations:** 1 Center for Functional Nanomaterials, Brookhaven National Laboratory, Upton, New York 11973, USA

## Abstract

Directed self-assembly (DSA) of block copolymers is an emergent technique for nano-lithography, but is limited in the range of structures possible in a single fabrication step. Here we expand on traditional DSA chemical patterning. A blend of lamellar- and cylinder-forming block copolymers assembles on specially designed surface chemical line gratings, leading to the simultaneous formation of coexisting ordered morphologies in separate areas of the substrate. The competing energetics of polymer chain distortions and chemical mismatch with the substrate grating bias the system towards either line/space or dot array patterns, depending on the pitch and linewidth of the prepattern. This is in contrast to the typical DSA, wherein assembly of a single-component block copolymer on chemical templates generates patterns of either lines/spaces (lamellar) or hexagonal dot arrays (cylinders). In our approach, the chemical template encodes desired local spatial arrangements of coexisting design motifs, self-assembled from a single, sophisticated resist.

## Abstract (web summary)

There is a limited range of structures available in nanolithography using directed self-assembled block copolymers. Here, Black and co-workers expand directed self-assembly chemical patterning by using a blend of lamellar and cylinder forming block copolymers on surface chemical line gratings.

## Graphical abstract

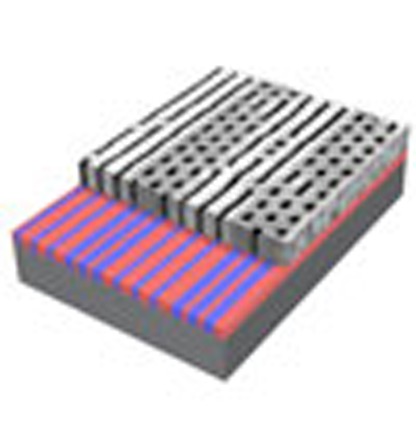

Directed self-assembly (DSA) is a nanofabrication technique in which self-assembling block copolymer thin films (BCP) are ordered using larger-scale, pre-defined guides patterned by standard lithography^1^,^2^,^3^. Typically, lamellar or cylindrical copolymer pattern morphologies are spatially templated by either topographic relief^4^,^5^,^6^,^7^ or patterned chemical inhomogeneity^8^,^9^,^10^ on the substrate. Commensurability between the guiding feature spacing and the natural pitch of the copolymer (*L*_0_) and between feature size and BCP domain size induces a high degree of positional and orientational order in the self-assembled, nanoscale patterns. Directed self-assembly is limited in the range of structures that can be fabricated in a single layer because each BCP material defines only a single equilibrium morphology. Previous work has shown that BCP patterning using two morphologies can be achieved through irradiation-induced BCP cross-linking and subsequent morphology alteration in non-cross-linked areas through solvent annealing^11^,^12^. However, these approaches to create mixed morphology patterns require multiple steps, potentially limiting the registration accuracy of the two morphologies. Non-bulk morphological phases based on surface reconstruction of very thin cylinder-forming BCP films may provide a route to achieving mixed morphological patterning in a single layer^13^, but utilizing this in DSA requires impeccable control over BCP film thickness across nanometre scale distances.

In this work, we describe a new approach that enforces coexistence of multiple, aligned block copolymer morphologies within a single patterning layer (Fig. 1b). In addition to enforcing positional order, here the template locally selects the desired motif of a block copolymer resist capable of forming multiple patterning morphologies. This extension to DSA involves furthering the role of the template guiding the assembly process.

## Results

### Assembly of single morphology BCP on grating prepatterns

**Figure 1:**
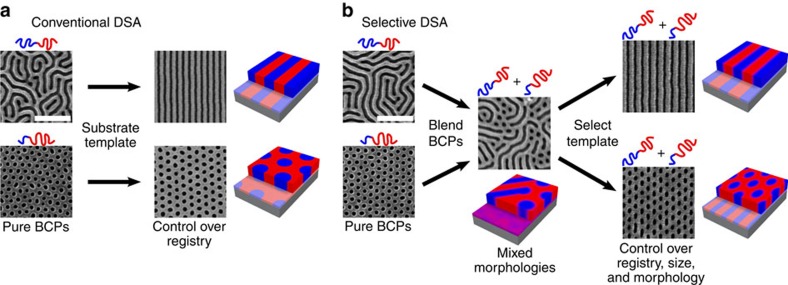
Conventional and selective directed self-assembly. (**a**) Directed self-assembly utilizes a substrate prepattern to impart long-range order to both lamellar and cylindrical self-assembled block copolymer films. (**b**) In selective directed self-assembly, the substrate prepattern also selects the local morphology formed by a block copolymer blend (either cylindrical or lamellar), in addition to imparting long-range order. White scale bar in scanning electron microscope images denotes 250 nm.

**Figure 2:**
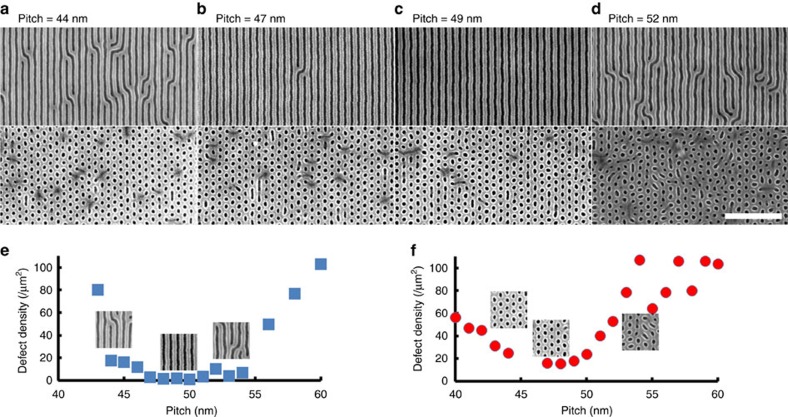
DSA of lamella and cylinders on chemical line grating patterns. (**a**–**d**) Scanning electron microscope images of lamella (top row) and cylinder (bottom row) forming block copolymers self-assembled on chemical line grating patterns with (**a**) 44 nm, (**b**) 47 nm, (**c**) 49 nm and (**d**) 52 nm. The white scale bar denotes 400 nm. (**e**,**f**) Defect density versus chemical pattern pitch for (**e**) lamellar and (**f**) cylindrical phase block copolymer films with representative scanning electron microscope images.

We advance upon conventional DSA chemo-epitaxy (Fig. 1a) by ordering sophisticated blend materials using chemical templates (Fig. 1b). Using electron beam lithography and O_2_ plasma etching, we generated chemically patterned substrates of alternating hydrophobic, polystyrene (PS) and hydrophilic (nominally SiO_2_) stripes, which imparts a high degree of translational order to the assembly of the individual lamellar polystyrene*-block*-poly(methyl methacrylate) (PS-*b*-PMMA) (*M*_W_=104 kg mol^–1^, PS:PMMA 50:50) (Fig. 2a–d top row) and cylindrical (*M*_W_=99 kg mol^–1^, PS:PMMA 54:46) (Fig. 2a–d bottom row) BCP phases. In practice, we fabricate multiple copies each grating prepattern, systematically increasing the electron beam exposure dose between copies to widen the linewidth of the hydrophilic oxide stripe while keeping the spacing constant. It is challenging to measure the absolute chemical pattern linewidths; nevertheless, exposure dose provides a robust means of systematically varying this width. The BCP morphologies align and register to the prepattern because the PMMA block lowers its energy by segregating to the SiO_2_ stripes, while the PS block wets PS substrate regions. We quantify the quality of the self-assembled pattern by calculating defect density at each pitch, using image analysis (Fig. 2e,f)^7^,^14^,^15^. The lamellar BCP remains highly ordered with almost zero defects across a range of chemical prepattern pitches, centred on the intrinsic BCP repeat period (*L*_0_=48 nm) (Fig. 2e) as one would expect for line-forming patterns on a line grating. The cylindrical morphology also aligns well with low defectivity, tolerating substantial mismatch between its intrinsic repeat period (for a cylindrical morphology, this is the distance between cylinder rows, *L*_0_=44 nm) and the prepattern pitch (Fig. 2f). The lamellar and cylinder systems exhibit some qualitative differences in their response to pattern mismatch (for example, sharpness of the transition from ordered to disordered patterns); we exploit this differential tolerance to locally select the pattern motif of a blended lamellar/cylindrical BCP resist.

### Assembly of BCP blend on grating prepatterns

**Figure 3:**
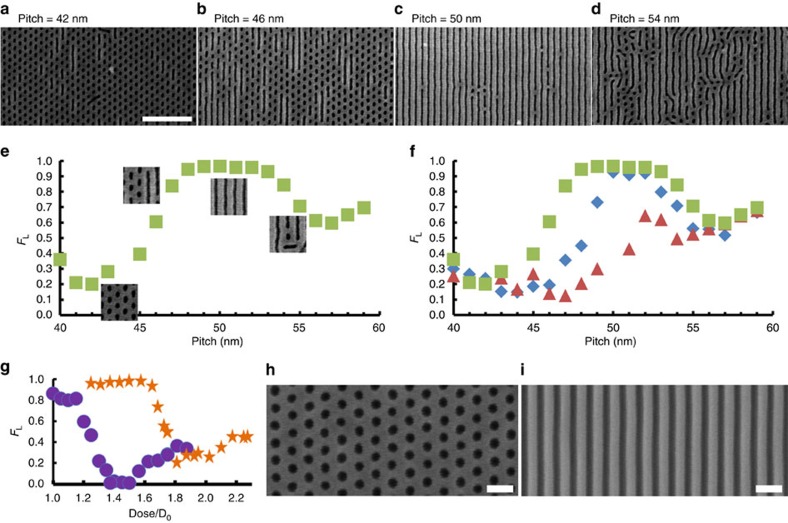
DSA of BCP blends on line gratings. (**a**–**d**) Scanning electron microscope images of a 1:1 lamella:cylinder blend self-assembled on chemical line grating patterns with (**a**) 42 nm, (**b**) 46 nm, (**c**) 50 nm and (**d**) 54 nm using an electron beam exposure dose of *D*=1.1 *D*_0_ (where *D*_0_=1,600 μC cm^−2^) to define the chemical pattern. The white scale bar denotes 400 nm. (**e**) Fractional pattern area covered by lines *F*_L_, versus pitch of the underlying chemical prepattern for a single electron-beam dose *D*=1.1 *D*_0_. *F*_L_=1 denotes an entire pattern of lines, *F*_L_=0 is completely dots. (**f**) Fractional pattern area covered by lines *F*_L_ versus chemical prepattern pitch for different electron-beam prepattern doses, 1.1 *D*_0_ (green squares), 1.2 *D*_0_ (blue diamonds) and 1.3 *D*_0_ (red triangles) (where *D*_0_=1,600 μC cm^−2^). For a given grating pitch, as we increase the dose, the resulting oxide stripes are wider. The process is optimized by selecting two pitches preferential for forming lines or dots and varying the dose. (**g**) *F*_L_ versus dose for prepattern pitch of 46 nm (purple circles) and 50 nm (orange stars). Parameters can be independently selected for *F*_L_=0 or 1 in a single exposure. In **h** and **i**, we utilize the optimal SiO_2_ width and pitch to select from the blend a single morphology, aligned and nearly defect-free overextended areas.

A thin film made from a 1:1 blend of the same lamellar and cylindrical block copolymers assembles differently on the same type of underlying line/space chemical prepatterns (Fig. 3a–d), forming well-ordered hexagonal cylinder patterns for some prepattern pitches (for example, 42 nm, Fig. 3a) and line/space patterns for others (for example, 50 nm, Fig. 3c). The BCP blend has an *L*_0_ of 46 nm when formed on a substrate with a random PS/PMMA polymer brush^16^ (Supplementary Fig. 1). We emphasize that in these experiments we do not observe macrophase separation of the blend components. Rather, the two BCP materials remain intimately mixed, as evidenced by the uniform patterns across the macroscopic sample dimensions (Fig. 3a–d). Cross-sectional SEM images show the films to be two-dimensional with a thickness of 27 nm, without evidence of vertical segregation of constituent chains, or a more complex three-dimensional structure (Supplementary Figs 2–4).

We quantify the fractional pattern area covered by lines (*F*_L_) by analysis of the SEM images, where *F*_L_=0 denotes a complete hexagonal dot pattern and *F*_L_=1 corresponds to entirely lines/spaces. In Fig. 3a–d, the directed pattern morphology is entirely lines/space (*F*_L_∼1) for grating prepattern pitches between ∼48 and 54 nm, and transitions sharply to a majority hexagonal dot array (*F*_L_∼0.2) for pitches <44 nm (Fig. 3e). Although the prepattern pitch is precisely controlled, there is greater uncertainty with respect to the oxide stripe width. This stripe width likely decreases slightly for large prepattern pitches due to the nature of the fabrication process. Chemical patterns with pitch larger than ∼55 nm result in poorly aligned patterns with mixed morphology (*F*_L_∼0.6). Thus, the displayed pattern morphology can be selected based on the pitch of the underlying chemical pattern.

Chemical prepatterns with the same set of pitches but fabricated using higher electron beam exposure create systematically wider SiO_2_ linewidths and wider hydrophilic regions (see blue and red symbols in Fig. 3f) and can change the type of pattern formed by the blend from lines to dots, as evidenced at the prepattern pitch of 48 nm, where the blend changes from forming nearly uniform lines/spaces (*F*_L_∼1, green squares in Fig. 3f) to hexagonal dots (*F*_L_∼0.2, red triangles in Fig. 3f) as the oxide stripe widens. We optimized the process by selecting single grating pitch parameters preferential for either line or dot patterns and varying the exposure dose to control the SiO_2_ linewidth. Figure 3g shows the line fraction for a 46 nm prepattern pitch (purple circles) and 50 nm prepattern pitch (orange stars). The parameters of pitch and oxide linewidth can be independently selected, making it possible to program regions of fully ordered, nearly defect-free hexagonal dot arrays with *F*_L_∼0 (Fig. 3h) and line gratings *F*_L_∼1 (Fig. 3i) within a single BCP blend on the same substrate.

Blends of block copolymers can form either homogeneous single phases, or coexisting phases^17^,^18^,^19^,^20^,^21^,^22^,^23^. We recently demonstrated that in thin films, blends of BCP cylinders and lamellae can either form a single-phase or two-phase morphologies^16^. Coexistence is a signature of the energy-degeneracy of two possible morphologies. In the present work, we exploit this phenomena, using a blend composition (1:1) designed to give coexistence of dots and lines on unpatterned, neutral substrates (Fig. 1b). Nevertheless, our ability to locally select the morphology through the underlying template pitch is surprising because one might naively expect lamellar morphologies to always be favoured (compared with hexagonal dot arrays) on chemical line/space patterns, given their symmetry match to the guiding pattern. Instead, we observe that certain ranges of template pitch and linewidths drive the blend to adopt a pattern of hexagonally registered dots.

### Understanding self-assembly of BCP blends

**Figure 4:**
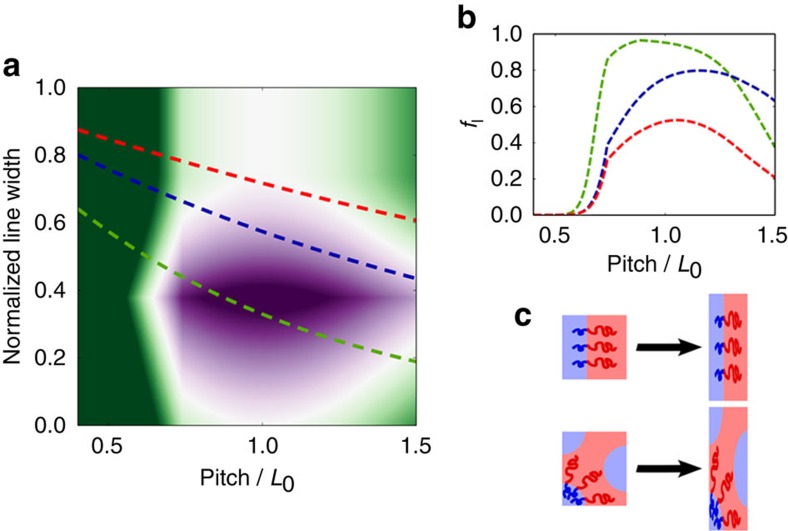
Predictions of an energy model for ordering of a BCP blend on a chemical line pattern. (**a**) Predicted relative energy between competing formation of line patterns and dot patterns, as a function of the pitch and the normalized oxide linewidth of the underlying chemical pattern. Purple indicates that line patterns are lower energy, while green indicates dot patterns are lower energy. White indicates that the two pattern types are energetically degenerate; one would thus expect to see coexistence of patterns. (**b**) Fraction of line patterns (*f*_l_) as a function of pitch, for three different doses used to generate the chemical pattern. A constant dose corresponds to a curve through the energy landscape, shown by the corresponding dashed lines in **a**. Depending on the pitch or dose, either line patterns or dot patterns will be favoured. Predictions are consistent with the experimental results of Fig. 3f. (**c**) Microscopic origin of the relative energies. When the template pitch deviates from the equilibrium spacing of the BCP pattern, the polymer chain must distort (compress or stretch) to accommodate. For line patterns, variation of pitch causes all the polymer chains to compress (or stretch). By comparison, the hexagonal-dot pattern, when distorted, involves a combination of chain stretching and compression (with some chains relatively unperturbed). Thus, dot patterns are more ‘tolerant' of distortion. (Refer to Supplementary Information for details).

We can understand this phenomenon by considering the balance between the energetics of chain distortions (stretching or compression), and the interfacial energy associated with polymer ordering on the chemical prepattern (Supplementary Figs 5–11 and Supplementary Discussion). Incommensurate chemical prepatterns distort the BCP unit cell, incurring an energy penalty due to polymer chain distortion^24^,^25^. These molecular distortions are different for lamellar versus cylindrical unit cells (Fig. 4c). For lamellae ordering, all polymer chains align along the lamellar normal, which is also along the chemical grating pitch vector, so that assembly on an incommensurate chemical pattern requires all chains to distort. In a hexagonal-cylinder unit cell, polymer chains are instead arranged in a 360° spread of orientations. Assembly of hexagonal cylinders on a chemical pattern with a mismatched pitch causes some chains to compress and others to stretch, while some chains remain relatively unperturbed (Fig. 4). This considerably lowers the overall energy penalty for distorting the hexagonal unit cell compared with that of the lamellar. We see clear evidence for distortion of the BCP morphologies on chemical patterns. The morphologies alter their repeat period to match the underlying pitch. In the case of dot patterns, the cylinder cores are distorted into ellipses.

At the same time, interfacial energies enforce strong wetting preferences (PMMA preferentially wetting SiO_2_; PS preferring wetting the PS brush). The hexagonal-dot morphology is intrinsically mismatched to a chemical grating prepattern, such that there is an interfacial mismatch, for all prepattern pitches and relative oxide stripe widths, creating a significant energy penalty.

Competition between the chain distortion and substrate interfacial energies drives the observed morphology selectivity, with hexagonal cylinder patterns more tolerant to deviations in chemical prepattern pitch and line patterns more tolerant to variations in oxide linewidth. When the chemical pattern pitch matches the natural repeat-spacing of the PS-*b*-PMMA blend, the line morphology is lower energy because it minimizes the interfacial mismatch. However, for incommensurate chemical patterns, the hexagonal dot pattern may become lower energy because it more readily accommodates the required, substantial chain distortions. The calculated energy landscape (Fig. 4a) displays a central region (purple) where both pitch and relative oxide stripe width match that of the blend, so that the material orders into a line pattern. Substantial deviations in prepattern pitch result in hexagonal dot patterns (green). In some regions of the phase diagram, the energy of these two states is nearly identical (white), which experimentally gives rise to coexistence of both pattern types. Different electron-beam exposure dose during the prepattern writing is equivalent to taking different slices through this energy landscape. Figure 4b shows the predicted morphology mixture for three different doses (the corresponding slices through the energy landscape are shown in Fig. 4a), demonstrating that the morphology makeup is strongly responsive to the underlying chemical pattern pitch and linewidth. These results can also be compared with the experimental data in Fig. 3f. The two sets of curve are in strong qualitative agreement. That is, theory and experiment both demonstrate that BCP blend morphologies can be tuned using chemical patterns. Furthermore, we can generate different local pitches and/or doses during inscription of the chemical pattern, in order to locally select different morphologies.

### Increasing complexity of self-assembled BCP blend

**Figure 5:**
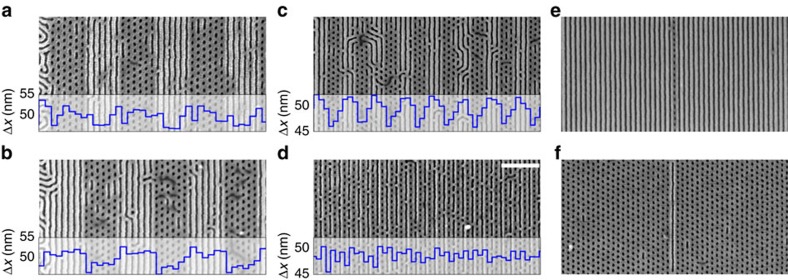
Selective directed self-assembly of simultaneous coexisting morphologies. Images have an overlay of the local morphology spacing (Δ*x*, blue line), across the grating pitch (computed using image analysis). By properly selecting line pitch and width, we can arbitrarily pattern regions with either dots or lines. The chemical patterns used in **a** and **b** have identical alternating regions of pitch=50 and 46 nm. The dose of each region has been independently selected such that the resulting oxide stripe width in the chemical pattern forces either dot or line patterns. In **c** and **d**, we see that selective DSA can generate alternating morphologies down to single columns of lines and dots in close proximity. Alternately, tuning the chemical prepattern can force the BCP blend to form a single column of dots in a field of lines (**e**) or line in a field of well-ordered dots (**f**).

The results imply that we may locally encode the pattern motif of a cylinder/lamellae BCP blend resist using pitch and/or oxide stripe width of the underlying chemical prepattern. Through careful design, we use this approach to generate arbitrary, localized morphologies within a single self-assembled copolymer blend layer. For example, grating prepatterns with pitch alternating between regions 50 and 46 nm can be programmed to assemble either alternating dot array/line array regions (Fig. 5a) or the inverse (Fig. 5b) by independently changing the widths of the oxide stripes within the two regions of the prepattern. By locally varying the pitch of the chemical prepattern, we can similarly program regions of hex dots and lines ranging from multiple periods of each to a single period. This is emphasized in Fig. 5 by the blue overlaid traces, which are measurements of the local spacing of the BCP morphology (Δ*x*), in the direction of the grating vector. For example, in Fig. 5b the regions where the prepattern has a larger pitch form a line morphology, with a correspondingly larger pitch; whereas regions with a tight prepattern pitch form a dot morphology with a small pitch. In the limit of directing the assembly of alternating single columns of lines and dots, we rely on the material's sensitivity to both prepattern pitch and the width of the oxide stripes to program the location of each (Fig. 5d). More complex, non-uniform areas of dots and lines can be programmed through suitable prepattern design such as an ordered single column of dots in an otherwise uniform line grating (Fig. 5e) or the inverse pattern consisting of a single line in a field of ordered dots (Fig. 5f) or even more arbitrary geometries (Supplementary Figs 12–18). Thus, selective directed self-assembly affords an additional means of controlling the ordering BCP layer: by locally selecting the morphology, in addition to the spatial registry and alignment afforded by patterning DSA approaches. This enables the resist to form structures at a length scale below the chemical prepattern: for example, the radius of curvature of the dot patterns is encoded in the BCP molecules, and not the directing template. A variety of clever and effective DSA template designs have been presented in the literature, where it has been demonstrated that one can locally redirect the morphology's orientation to generate more sophisticated patterns^11^,^26^,^27^,^28^,^29^,^30^,^31^. An exciting opportunity for future research is combining this selective DSA methodology with more advanced template types, as they afford complementary control over local ordering.

## Discussion

We have demonstrated local control of the morphology of a self-assembling BCP blend using chemical patterns, selectively forming either line patterns or dot patterns by locally adjusting the relative energy of these competing morphologies through spatial variations of the underlying chemical pattern. This responsive resist thus enables selective DSA, wherein the chemical template not only controls the registry of the resist morphology, but also selects among different possible local nanoscale ordering states. Selective DSA is a generalized method, wherein multi-component blends of self-assembling materials are ordered in the presence of spatially varying directing field. The directing forces are thus exploited to locally control nanoscale pattern formation.

## Methods

### Sample fabrication

Silicon chips (∼1 cm^2^) were cleaned in O_2_ plasma and then coated with a hydroxyl-terminated polystyrene brush (*M*_W_=11 kg mol^−1^) (Polymer Source, Inc.) by spin-casting from toluene (1% wt) and annealing at 200 °C for 4 h in a vacuum oven. Unattached PS was removed after annealing by rinsing with toluene. The advancing contact angle of the prepared PS brush was measured at 94±6° and a receding contact angle of 77±5°. PMMA electron-beam resist was spin-coated to a thickness of ∼50 nm and baked on a hot plate at 180 °C for 3 min. Line/space grating patterns were exposed in a JEOL JBX6300-FS electron beam lithography tool using 1 nA beam current with doses ranging from 1,200 to 2,080 μC cm^−2^. After exposure, the samples were developed in room temperature methyl isobutyl ketone: isopropyl alcohol (IPA)(1:3) for 60 s and rinsed in isopropyl alcohol.

Exposed grating patterns were transferred to the PS brush by oxygen plasma etching (March Plasma CS1701) RIE tool using 82 mTorr O_2_, 14 W radiofrequency power for ∼30 s. The remaining PMMA was removed by soaking in toluene at 60 °C for 10 min, with the final 5 min in an ultrasonic bath. Block copolymer solutions (Polymer Source, Inc.) consisted of lamellar-forming material (*M*_W_=104 kg mol^−1^, polydispersity 1.09, 50.1% PS content) and cylinder-forming material (*M*_W_=99 kg mol^−1^, polydispersity 1.09, 63.8% PS), at 1% concentration in toluene. We performed gel permeation chromatography (GPC) and nuclear magnetic resonance (NMR) to confirm material purity (Supplementary Figs 19–22). BCP films were deposited by spin-coating at 2,000 r.p.m. for 45 s. The films were thermally annealed in a vacuum oven at 205 °C for ∼12 h.

To increase the contrast during SEM imaging, after anneal, samples were illuminated with ultraviolet light for 5 min and developed in acetic acid to remove the PMMA block. Images of uncoated samples were taken with an Hitachi S-4800 scanning electron microscope with an accelerating voltage at 1 kV in deceleration mode. Data in Fig. 3 were obtained from a single chip simultaneously patterned with a range of grating pitch and exposure dose. We have imaged ∼10 of samples with similar results.

### Image analysis

We quantify order in the BCP patterns using image analysis of SEM data, as previously described in the literature^7^,^14^,^15^,^32^. We used code written in the Python programming language, exploiting libraries for image manipulation (Python Image Library), numerical computations (numpy)^33^ and plotting (matplotlib)^34^. For dot patterns, image thresholding and segmentation is used to identify the cylinder cores. The local topology of the hexagonal lattice is assessed by connecting cylinder cores to nearest neighbours, using a cutoff distance of 1.36 × the average cylinder–cylinder distance (that is, halfway between the first and second ‘rings' of cylinders surrounding a representative cylinder). For each cylinder, the number of neighbours (number of cylinders within the cutoff distance) is calculated. Using this nearest-neighbour map, defects can be identified by cylinders that deviate from the ideal state of having six nearest neighbours (regions where a cylinder is ‘missing' are surrounded by cylinders of five nearest neighbours, distortions of the hex lattice lead to local configurations where the nearest-neighbour count is larger or smaller than six, and so on). The areal defect density is computed by dividing the defect count by the image size (cylinders at the periphery of the image are ignored, since they lack the correct nearest-neighbour count owing to the image edge). For line patterns, image thresholding and segmentation is used to identify the endpoints of individual lines. Endpoints (other than at the image edge) correspond to local defects in the morphology. The trend of the areal defect densities provides a robust measure of the relative ordering quality of different samples. The relative areal coverage of line patterns (*F*_*L*_) versus dot patterns was assessed using particle counting^16^. A thresholded image was used to identify ‘particles' within the image, and a size cutoff was used to distinguish between regions of dots (small particles) and lines (large, elongated particles). The spacing between rows of aligned BCP morphology was assessed by summing pixel grayscale intensity values in each column in the SEM image. Images were first Fourier-filtered to suppress imaging noise, and rotated to align the experimental patterns with the pixel grid. Columns were summed to yield a one-dimensional oscillating curve. Peak-fitting (Gaussian fit constrained to a local part of the curve) was used to identify the positions of local maxima; distances between adjacent maxima were used to measure the local pattern spacing (Δ*x*).

### Data availability

All relevant data is available from the authors.

## Additional information

**How to cite this article:** Stein, A. *et al*. Selective directed self-assembly of coexisting morphologies using block copolymer blends. *Nat. Commun.* 7:12366 doi: 10.1038/ncomms12366 (2016).

## Supplementary Material

Supplementary Information Supplementary Figures 1-22, Supplementary Discussion and Supplementary References.

## References

[b1] Cheng J. Y., Ross C. A., Smith H. I. & Thomas E. L. Templated self-assembly of block copolymers: top-down helps bottom-up. Adv. Mater. 18, 2505–2521 (2006).

[b2] Koo K., Ahn H., Kim S.-W., Ryu D. Y. & Russell T. P. Directed self-assembly of block copolymers in the extreme: guiding microdomains from the small to the large. Soft Matter 9, 9059–9071 (2013).

[b3] Stein G. E., Mahadevapuram N. & Mitra I. Controlling interfacial interactions for directed self assembly of block copolymers. J. Polym. Sci. B Polym. Phys. 53, 96–102 (2014).

[b4] Bita I. . Graphoepitaxy of self-assembled block copolymers on two-dimensional periodic patterned templates. Science 321, 939–943 (2008). 18703736 10.1126/science.1159352

[b5] Segalman R. A., Hexemer A. & Kramer E. J. Effects of lateral confinement on order in spherical domain block copolymer thin films. Macromolecules 36, 6831–6839 (2003).

[b6] Sundrani D., Darling S. B. & Sibener S. J. Guiding polymers to perfection: macroscopic alignment of nanoscale domains. Nano Lett. 4, 273–276 (2004).

[b7] Yager K. G. . Evolution of block-copolymer order through a moving thermal zone. Soft Matter 6, 92–99 (2010).

[b8] Edwards E. W. . Dimensions and shapes of block copolymer domains assembled on lithographically defined chemically patterned substrates. Macromolecules 40, 90–96 (2007).

[b9] Edwards E. W., Stoykovich M. P., Solak H. H. & Nealey P. F. Long-range order and orientation of cylinder-forming block copolymers on chemically nanopatterned striped surfaces. Macromolecules 39, 3598–3607 (2006).

[b10] Ruiz R. . Density multiplication and improved lithography by directed block copolymer assembly. Science 321, 936–939 (2008). 18703735 10.1126/science.1157626

[b11] Son J. G., Chang J.-B., Berggren K. K. & Ross C. A. Assembly of sub-10-nm block copolymer patterns with mixed morphology and period using electron irradiation and solvent annealing. Nano Lett. 11, 5079–5084 (2011). 21992516 10.1021/nl203445h

[b12] Bosworth J. K., Black C. T. & Ober C. K. Selective area control of self-assembled pattern architecture using a lithographically patternable block copolymer. ACS Nano 3, 1761–1766 (2009). 19534477 10.1021/nn900343u

[b13] Knoll A. . Phase behavior in thin films of cylinder-forming block copolymers. Phys. Rev. Lett. 89, 035501 (2002). 12144400 10.1103/PhysRevLett.89.035501

[b14] Harrison C. . Dynamics of pattern coarsening in a two-dimensional smectic system. Phys. Rev. E 66, 011706 (2002). 10.1103/PhysRevE.66.011706 12241374

[b15] Majewski P. W. & Yager K. G. Millisecond ordering of block copolymer films via photothermal gradients. ACS Nano 9, 3896–3906 (2015). 25763534 10.1021/nn5071827

[b16] Yager K. G., Lai E. & Black C. T. Self-assembled phases of block copolymer blend thin films. ACS Nano 8, 10582–10588 (2014). 25285733 10.1021/nn504977r

[b17] Abetz V. & Goldacker T. Formation of superlattices via blending of block copolymers. Macromol. Rapid Commun. 21, 16–34 (2000).

[b18] Hadziioannou G. & Skoulios A. Structural study of mixtures of styrene isoprene two- and three-block copolymers. Macromolecules 15, 267–271 (1982).

[b19] Hashimoto T., Koizumi S. & Hasegawa H. Ordered structure in blends of block copolymers.2. Self-assembly for immiscible lamella-forming copolymers. Macromolecules 27, 1562–1570 (1994).

[b20] Koizumi S., Hasegawa H. & Hashimoto T. Ordered structure in blends of block copolymers. 3. Self-assembly in blends of sphere- or cylinder-forming copolymers. Macromolecules 27, 4371–4381 (1994).

[b21] Matsen M. W. Immiscibility of large and small symmetric diblock copolymers. J. Chem. Phys. 103, 3268–3271 (1995).

[b22] Matsen M. W. & Bates F. S. One-component approximation for binary diblock copolymer blends. Macromolecules 28, 7298–7300 (1995).

[b23] Zhao J. . Phase behavior of pure diblocks and binary diblock blends of poly(ethylene)-poly(ethylethylene). Macromolecules 29, 1204–1215 (1996).

[b24] Hsu H.-P. & Grassberger P. Polymers confined between two parallel plane walls. J. Chem. Phys. 120, 2034–2041 (2004). 15268339 10.1063/1.1636454

[b25] Skvortsov A. M., Klushin L. I. & Birshtein T. M. Stretching and compression of a macromolecule under different modes of mechanical manupulations. Polym. Sci. Ser. A 51, 469–491 (2009).

[b26] Stoykovich M. P. . Directed self-assembly of block copolymers for nanolithography: fabrication of isolated features and essential integrated circuit geometries. ACS Nano 1, 168–175 (2007). 19206647 10.1021/nn700164p

[b27] Chang J.-B. . Design rules for self-assembled block copolymer patterns using tiled templates. Nat. Commun. 5, 3305 (2014). 24531135 10.1038/ncomms4305

[b28] Doerk G. S. . Enabling complex nanoscale pattern customization using directed self-assembly. Nat. Commun. 5, 5805 (2014). 25512171 10.1038/ncomms6805

[b29] Tavakkoli K. G. A. . Templating three-dimensional self-assembled structures in bilayer block copolymer films. Science 336, 1294–1298 (2012). 22679094 10.1126/science.1218437

[b30] Tavakkoli K. G. A. . Sacrificial-post templating method for block copolymer self-assembly. Small 10, 493–499 (2013). 23839974 10.1002/smll.201301066

[b31] Yang J. K. W. . Complex self-assembled patterns using sparse commensurate templates with locally varying motifs. Nat. Nanotechnol. 5, 256–260 (2010). 20228786 10.1038/nnano.2010.30

[b32] Berry B. C., Bosse A. W., Douglas J. F., Jones R. L. & Karim A. Orientational order in block copolymer films zone annealed below the order-disorder transition temperature. Nano Lett. 7, 2789–2794 (2007). 17691851 10.1021/nl071354s

[b33] Oliphant T. E. Python for scientific computing. Comput. Sci. Eng. 9, 10–20 (2007).

[b34] Hunter J. D. Matplotlib: A 2D graphics environment. Comput. Sci. Eng. 9, 90–95 (2007).

