# Supplementary material for: Selective directed self-assembly of coexisting morphologies using block copolymer blends
Source: Nat Commun. 2016 Aug 2;7:12366. doi: 10.1038/ncomms12366 (PMC4974660; doi:10.1038/ncomms12366)
Supplement: Supplementary Information — Supplementary Figures 1-22, Supplementary Discussion and Supplementary References. [file ncomms12366-s1.pdf]

Supplementary Figures

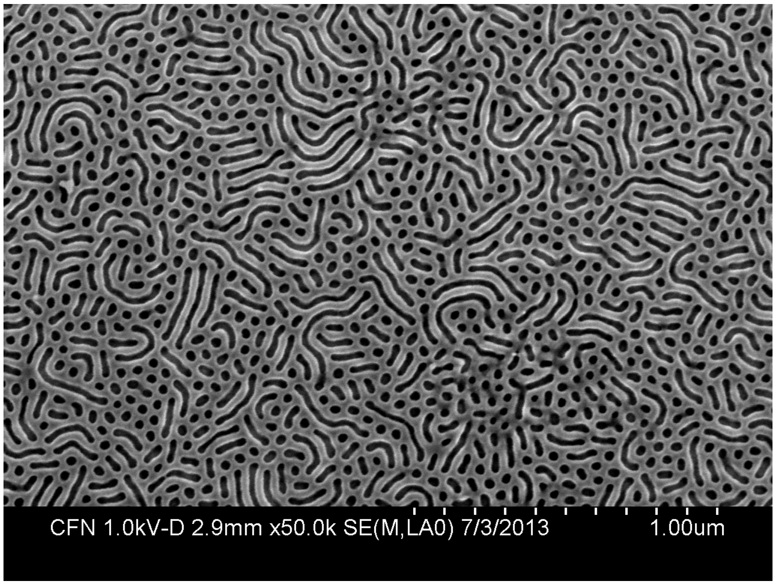

Supplementary Figure 1: SEM of the ordering of a block copolymer (BCP) blend (1:1 ratio of cylindrical-forming and lamellar-forming material) on a uniform (unpatterned) substrate. Image analysis indicates that the fractional area of line-patterns if $F_L \approx 0.7$, with an in-plane correlation length of $\xi \approx 60\,\mu$m, and a repeat-spacing of $L_0 = 46\,$nm.

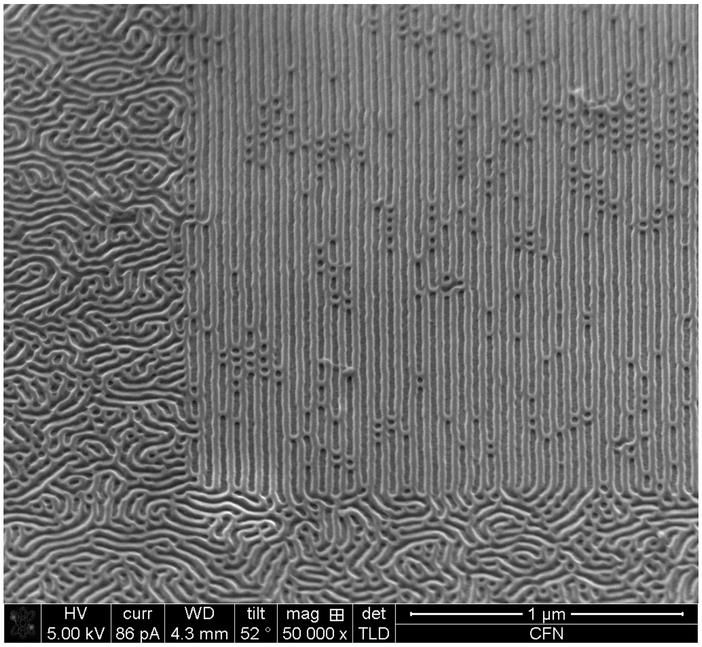

Supplementary Figure 2: Tilt-view (52°) SEM of the ordering of a block copolymer (BCP) blend (1:1 ratio of cylindrical-forming and lamellar-forming material). The region in the upper-right of the image had an underlying chemical pattern, resulting in alignment and registry of the BCP morphology.

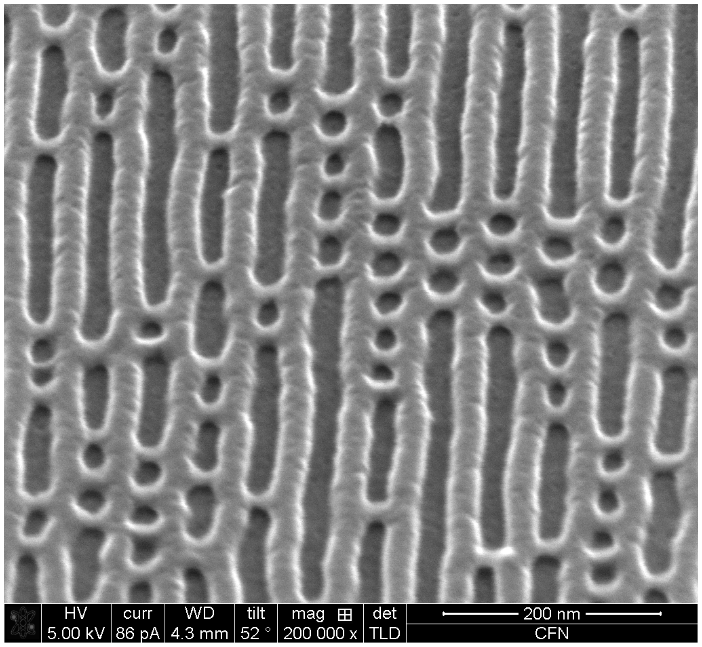

Supplementary Figure 3: Tilt-view (52°) SEM of a BCP blend ordering on a chemical line pattern. The relief shows no evidence of vertical structuring of the BCP morphology.

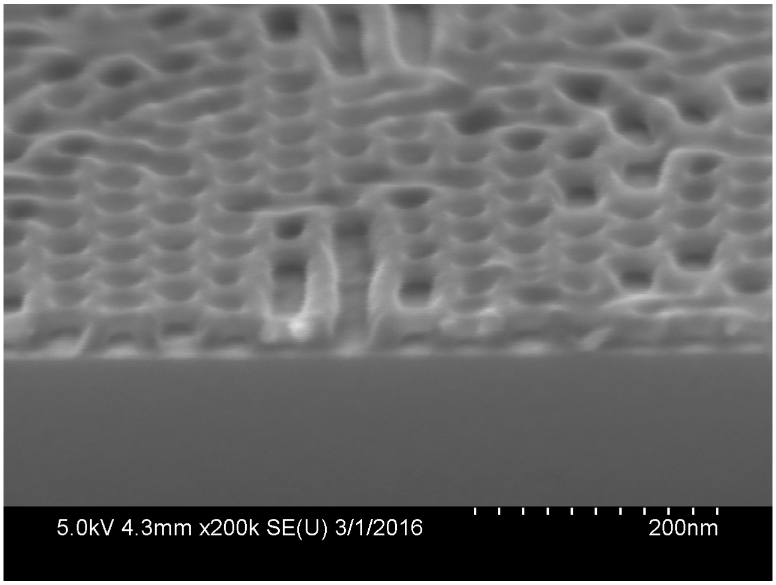

Supplementary Figure 4: Cross-section view (70°) SEM cross-section of a BCP blend ordering on a chemical line pattern. The relief shows no evidence of vertical structuring of the BCP morphology. The film thickness was measured to be 27 nm.

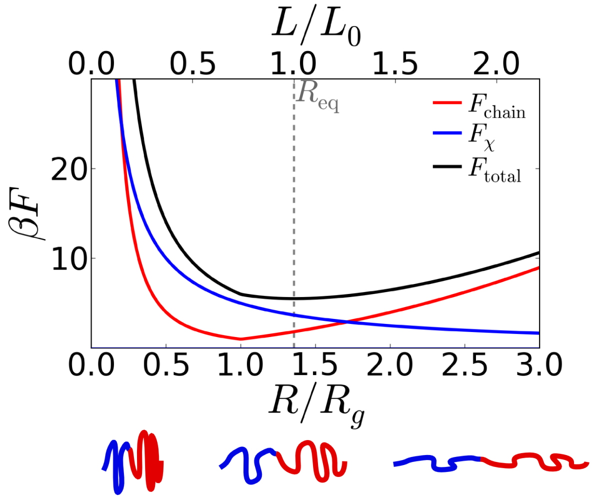

Supplementary Figure 5: Energy contributions for distortion of a polymer chain in a BCP morphology.

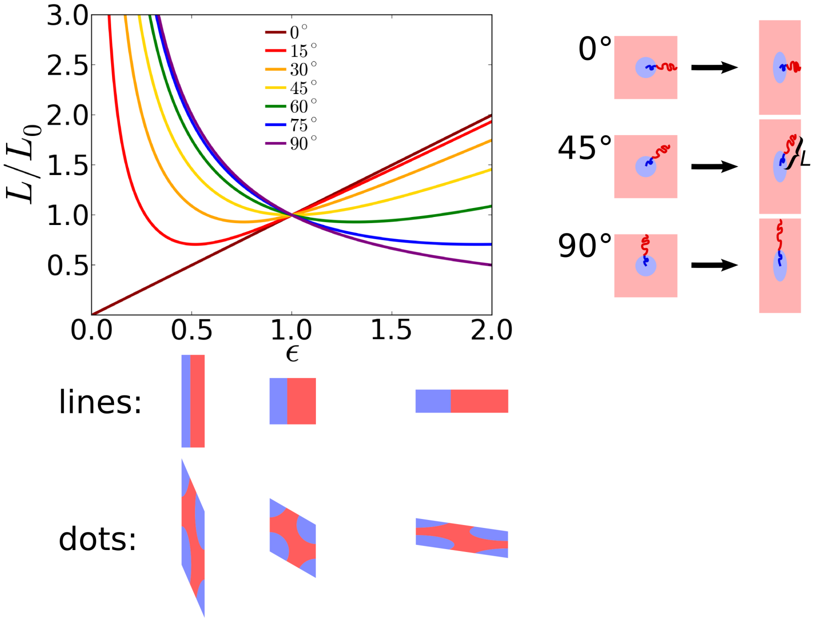

Supplementary Figure 6: Distortion of a BCP unit cell to an amount $\epsilon = L_{\text{pat}}/L_0$ correspondingly distorts the polymer chains to L/L_0. The amount of chain distortion (along the chain axis) depends on the orientation of the chain within the unit-cell.

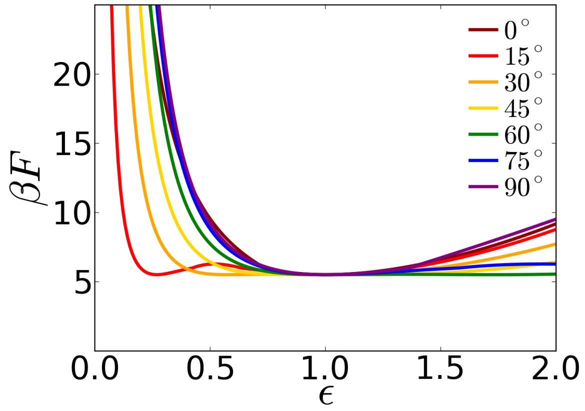

Supplementary Figure 7: Energy penalties for chains within a BCP morphology distorted to extent ϵ. The energy depends on the orientation of the chain within the morphology.

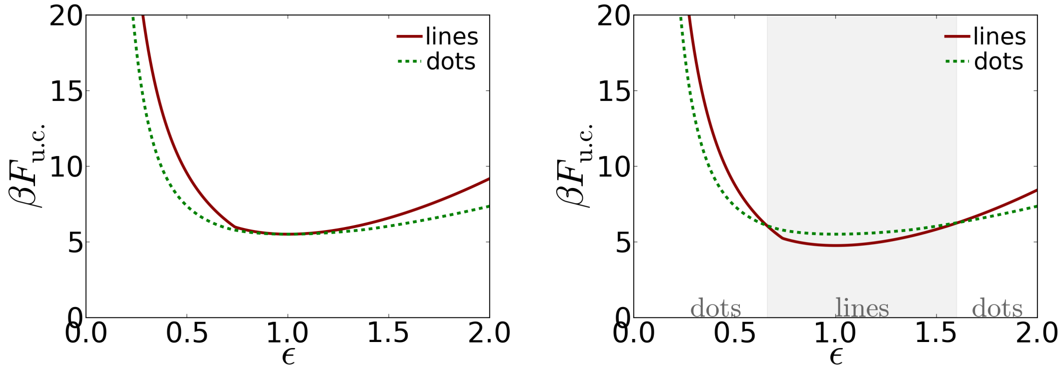

Supplementary Figure 8: (left) Overall energy for a BCP unit-cell exhibiting line or dot morphology, based on distorting the cell to extent ϵ. (right) Inclusion of an energy contribution from substrate-interaction shifts the two curves relative to one another. In some regimes, the line-patterns are lower energy, while in other regimes it is the dot-patterns.

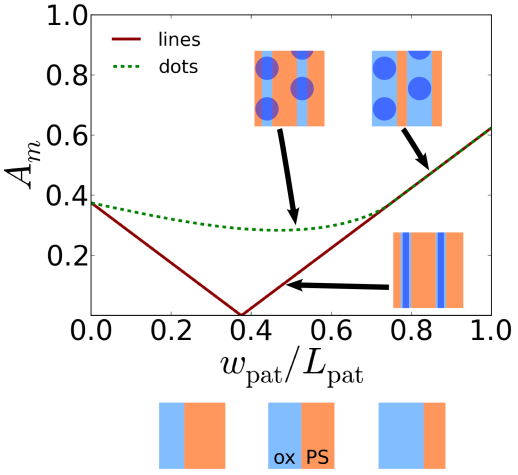

Supplementary Figure 9: Relative mismatch area (A_m) for dot and line morphologies sitting on stripe patterns. The line-patterns reach a minimum when their width exactly matches the duty-cycle ($w_{\mathrm{pat}}/L_{\mathrm{pat}}$). For dots, the energy penalty is generally larger, since the chemical stripes never exactly match the hexagonally-arranged dots.

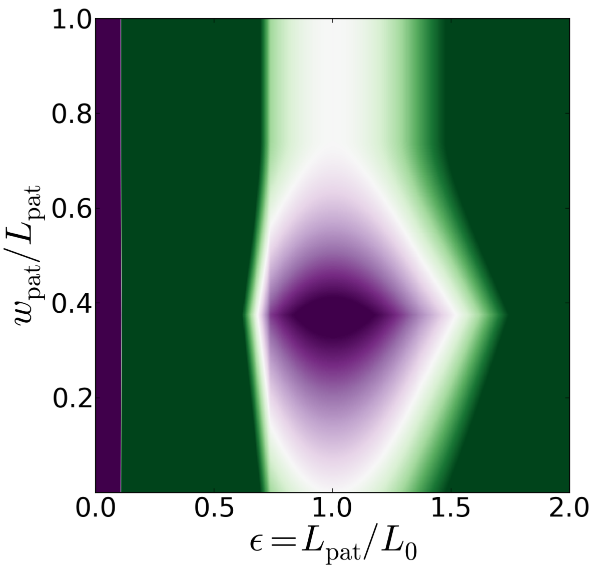

Supplementary Figure 10: Energy difference between dot and line-patterns for a BCP blend on a chemical stripe pattern. The y-axis is the duty-cycle of the pattern. The x-axis is the pitch of the pattern. The color scale is as follows: purple when line-patterns are lower energy, green when dot-patterns are lower energy, and white when the two phases have equal energy. Thus, in the central region, one expects to observe line-patterns. In the white regions, mixed morphologies (lines and dots) are likely. In the outer green regions, dot-patterns should be strongly preferred.

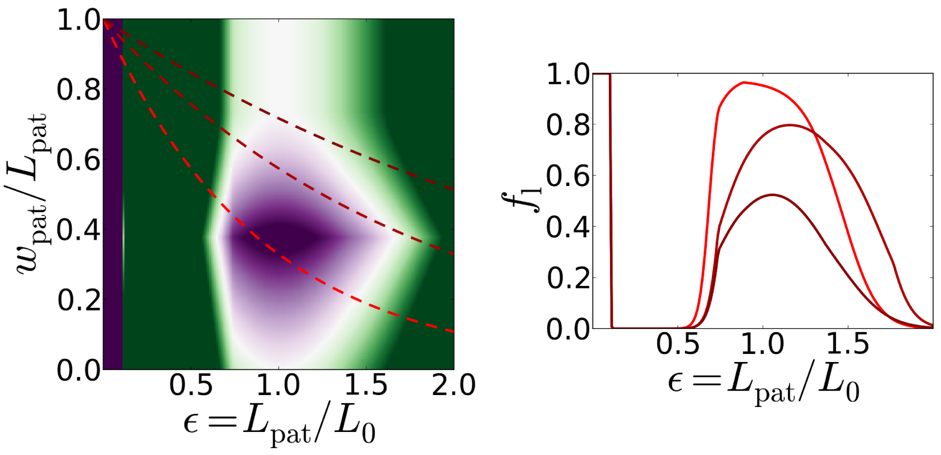

Supplementary Figure 11: Experimentally, chemical patterns with different dose are nonlinear traces through the phase diagram (left). Three different possible traces are shown (red dashed lines), representing different doses. The corresponding prediction for the areal fraction of line-patterns is also shown (right).

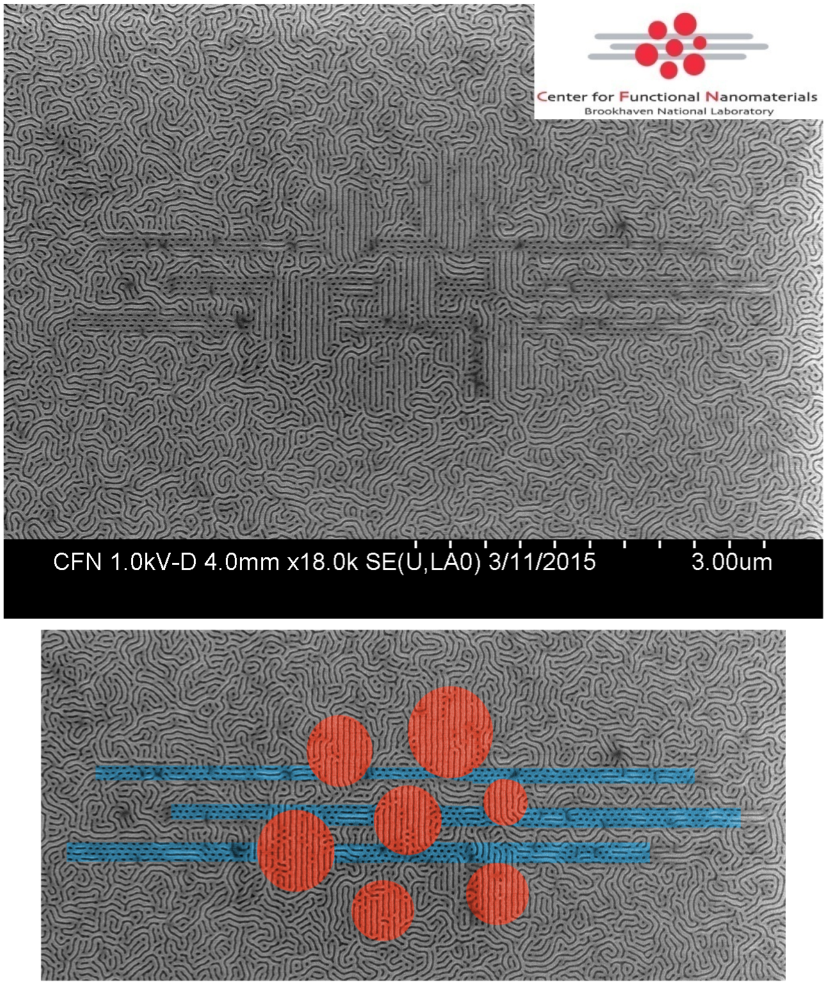

Supplementary Figure 12: SEM of a non-trivial chemical pattern, used to locally direct the registry and morphology of a BCP blend. The inset (upper right) shows the logo used as the design target. The lower SEM is color-coded to emphasize the design of the guiding chemical template.

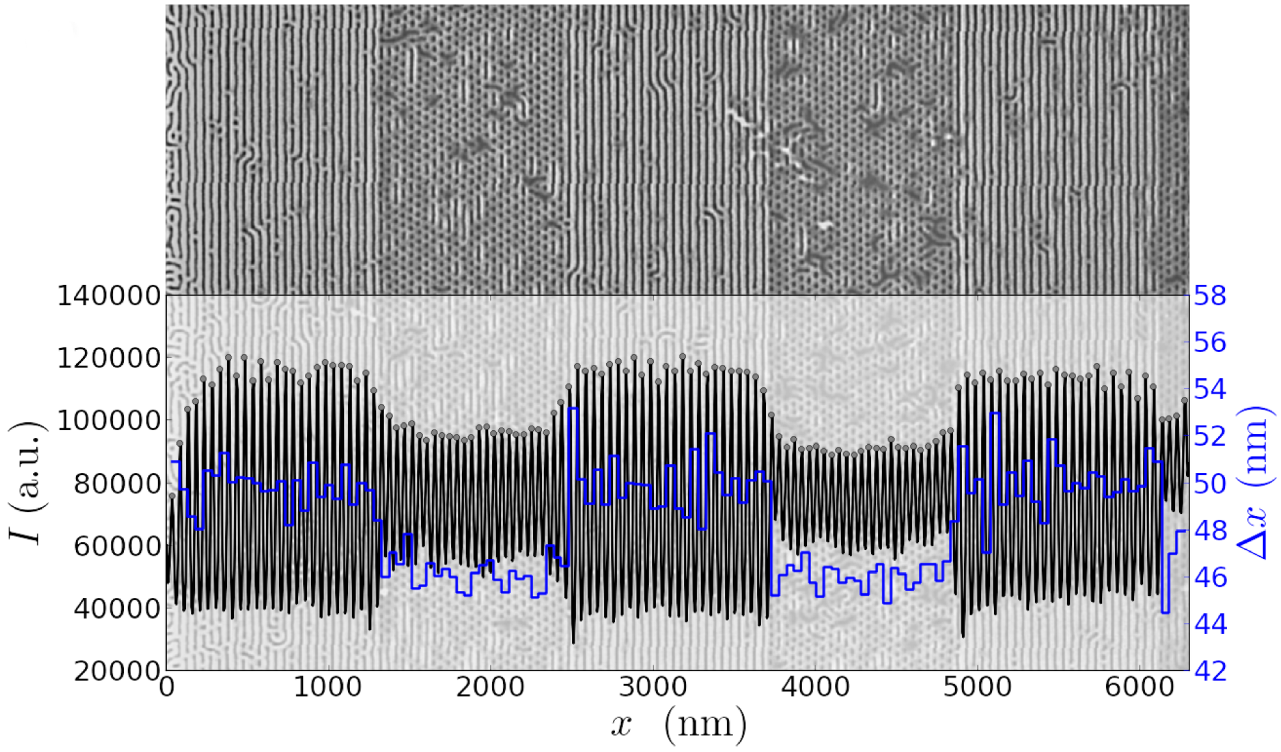

Supplementary Figure 13: SEM analysis of a BCP blend ordering on chemical line pattern, with regions of different template spacing giving rise to different BCP morphology. Image analysis is used to sum the image intensity along the columns (black curve), which is then converted into a measure of the local distance between neighboring rows of BCP morphology (blue line). As can be seen, regions of larger local pitch give rise to line patterns, whereas regions of smaller pitch form dot patterns.

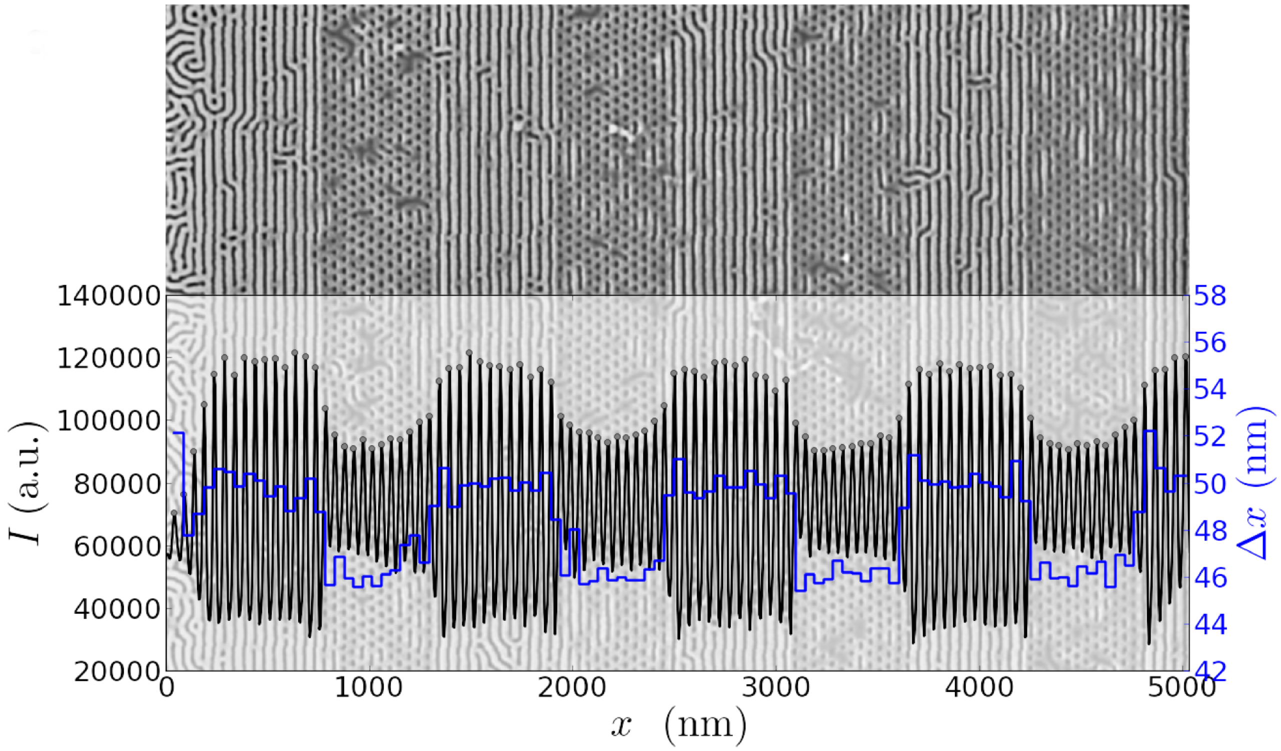

Supplementary Figure 14: SEM analysis of a BCP blend ordering on chemical line pattern, with regions of different template spacing giving rise to different BCP morphology.

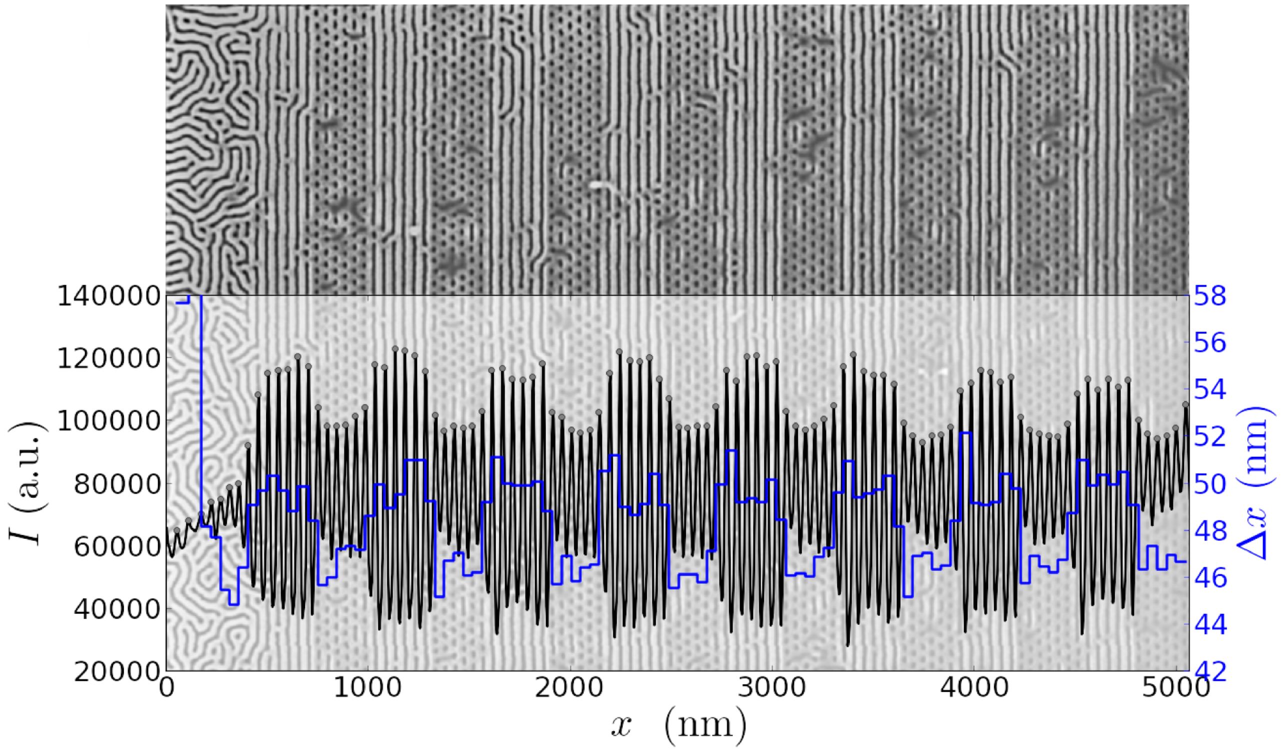

Supplementary Figure 15: SEM analysis of a BCP blend ordering on chemical line pattern, with regions of different template spacing giving rise to different BCP morphology.

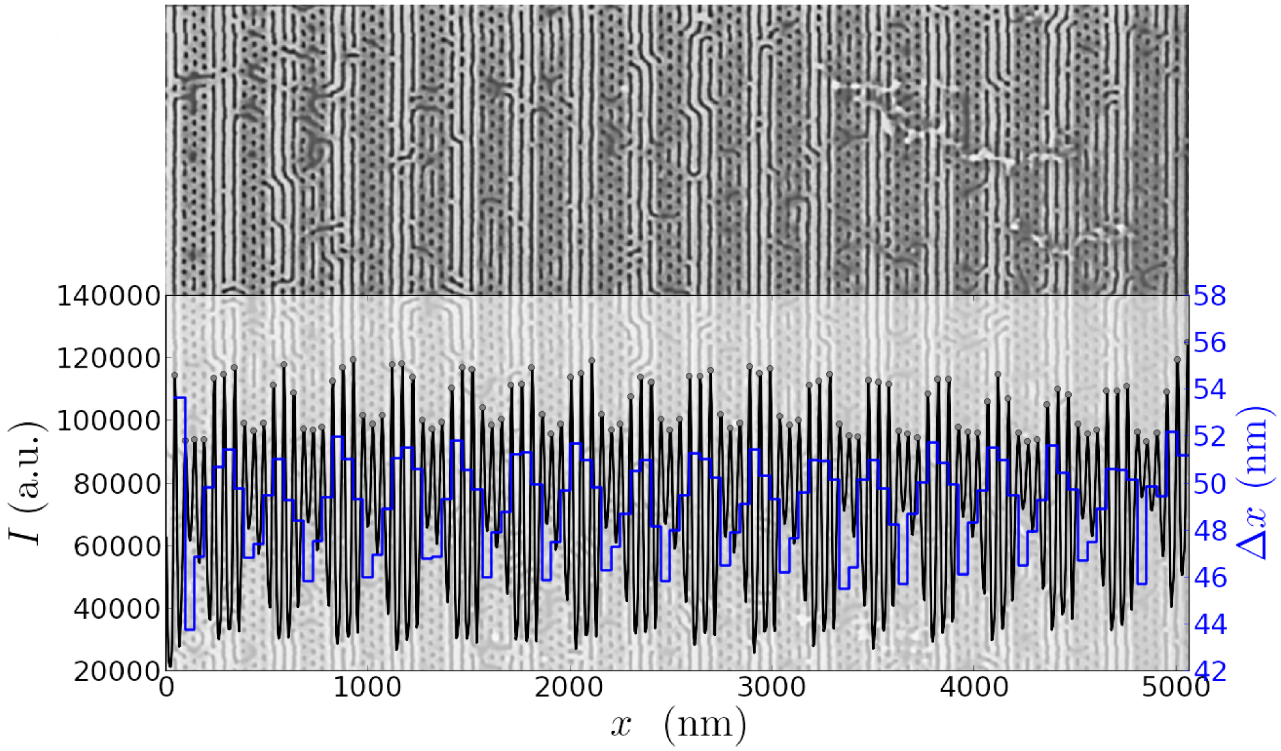

Supplementary Figure 16: SEM analysis of a BCP blend ordering on chemical line pattern, with regions of different template spacing giving rise to different BCP morphology.

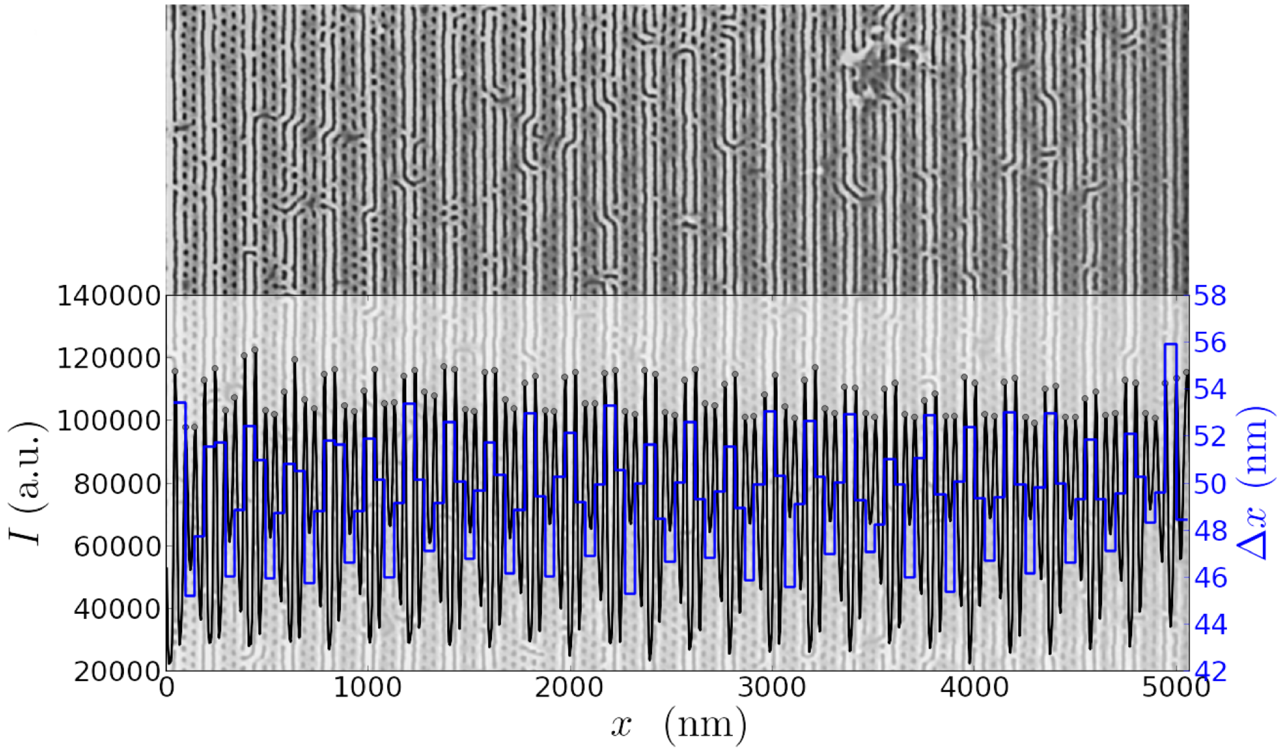

Supplementary Figure 17: SEM analysis of a BCP blend ordering on chemical line pattern, with regions of different template spacing giving rise to different BCP morphology.

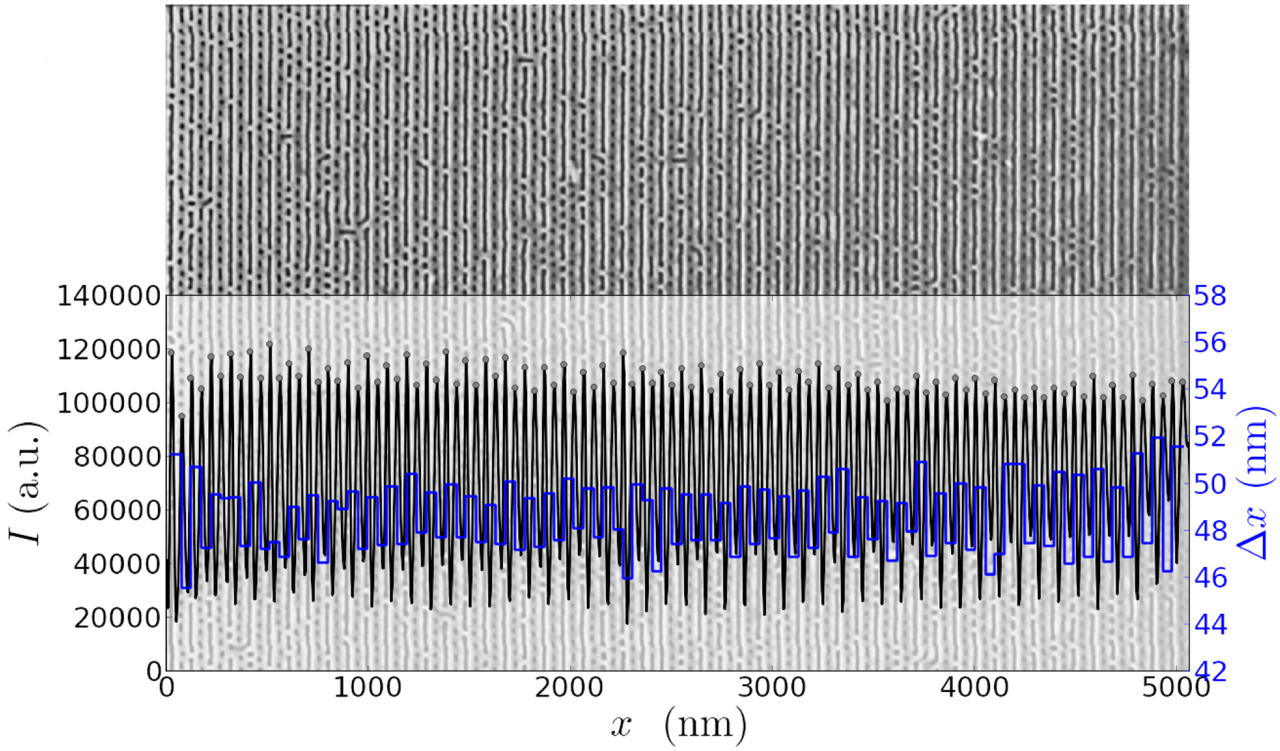

Supplementary Figure 18: SEM analysis of a BCP blend ordering on chemical line pattern. Alternating lines were exposed with slightly different electron dose. The alternating of patterning line width templates different BCP moprhologies.

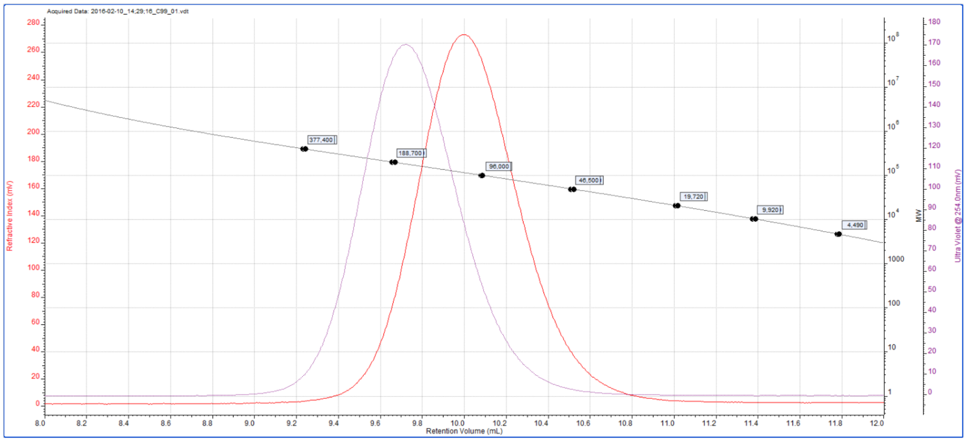

Supplementary Figure 19: Gel permeation chromatography (GPC) of cylinder-forming block copolymer material (99 kg/mol, polydispersity 1.09). The measurement uses both a UV detector (254 nm), and a differential refractive-index detection (in $CHCl_3$, $n = 1.44$). The former is sensitive only to the PS block, while the later responds to both PS ($n = 1.59$) and PMMA ($n = 1.49$). We measure a PS content of 63.8%, and find no evidence of contaminants.

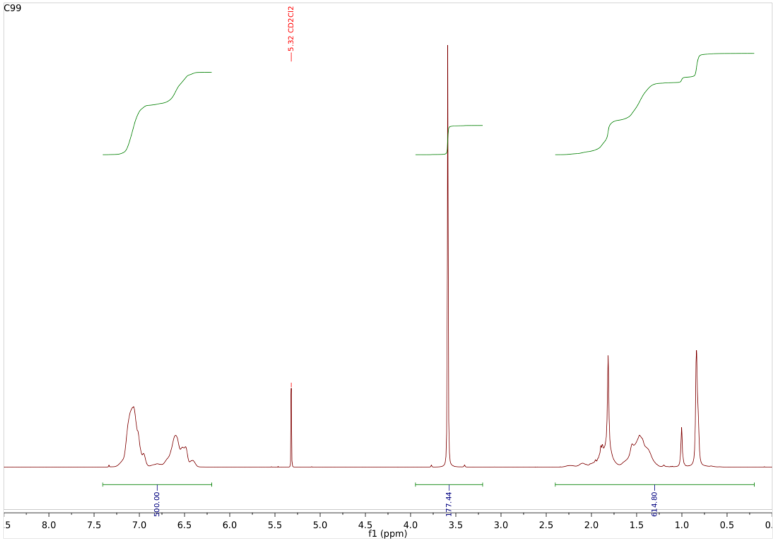

Supplementary Figure 20: Nuclear magnetic resonance (NMR) spectroscopy measurements of cylinder-forming block copolymer material (99 kg/mol, polydispersity 1.09); obtained in CD_2Cl_2 at 400 MHz. The data is consistent with a PS-*b*-PMMA diblock copolymer without other contaminants.

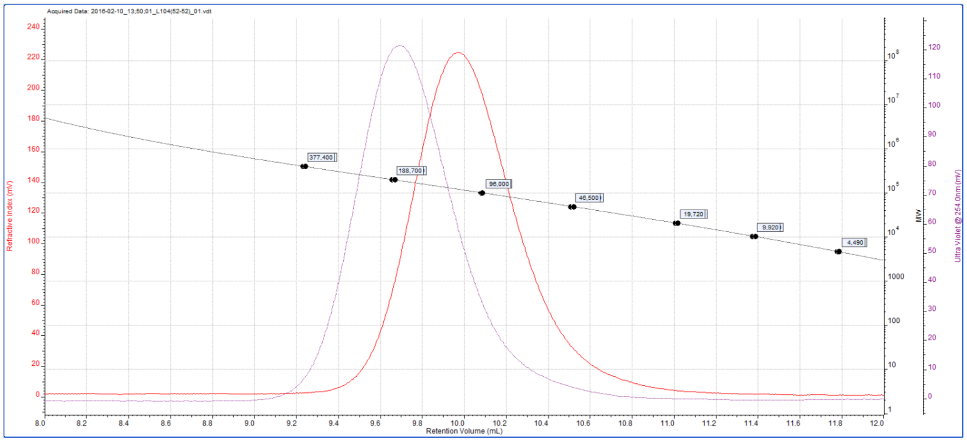

Supplementary Figure 21: Gel permeation chromatography (GPC) of lamellar-forming block copolymer material (104 kg/mol, polydispersity 1.09). The measurement uses both a UV detector (254 nm), and a differential refractive-index detection (in $CHCl_3$, $n = 1.44$). The former is sensitive only to the PS block, while the later responds to both PS ($n = 1.59$) and PMMA ($n = 1.49$). We measure a PS content of 50.1%, and find no evidence of contaminants.

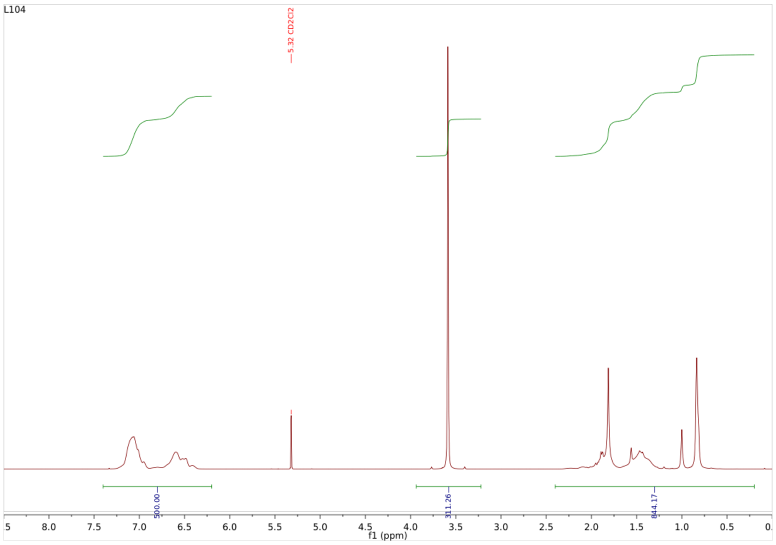

Supplementary Figure 22: Nuclear magnetic resonance (NMR) spectroscopy measurements of lamellar-forming block copolymer material (104 kg/mol, polydispersity 1.09); obtained in CD_2Cl_2 at 400 MHz. The data is consistent with a PS-*b*-PMMA diblock copolymer without other contaminants.

Supplementary Discussion

We present a simple predictive model for the ordering of BCP Blends on chemical stripe patterns.

BCP Blends

Consider a blend of two block-copolymers: a cylinder-forming material (CYL) and a lamellar-forming material (LAM). For concreteness, we consider the case where the matrix material is polystyrene (PS, denoted S), and the minority material is poly(methyl methacrylate) (PMMA, denoted M). If we blend the materials in a ratio of $\phi_{\mathrm{CYL}} = 1 - \phi_{\mathrm{LAM}}$, then we expect the final volume-fractions to be:

$$f_{\mathrm{Mb}} = f_{\mathrm{M,CYL}} \times \phi_{\mathrm{CYL}} + f_{\mathrm{M,LAM}} \times (1 - \phi_{\mathrm{CYL}}) \tag{1}$$

where the b stand for 'blend.' For lamellae, $f_{\mathrm{M,LAM}} \approx 0.50$; while for cylinders $f_{\mathrm{M,CYL}} \approx 0.25$. Thus for an equal-weighted blend ($\phi_{\mathrm{CYL}} = 0.50$), we expect $f_{\mathrm{Mb}} \approx 0.375$. Previous work has shown that such a BCP blend can exhibit different morphologies: (1) alternating lamellae, (2) hexagonally-packed cylinders, (3) mixture of both. Note that the lamelar-like pattern (1) is not identical to the unblended LAM phase, and the cylinder-like pattern (2) is not identical to the CYL phase. In particular, the blended phases must have a different volume-fraction of the matrix and minority components, to satisfy mass conservation. Thus, we instead refer to (1) as 'lines' and (2) as 'dots'. For the blending suggested above, the material can thus choose between 'thin lines' (i.e. a lamellar-pattern where the PMMA regions are thinner than the PS regions) or 'large dots' (i.e. hexagonally-arranged cylinders, where the cylinder-cores are larger than in the pure CYL phase).

Lines: For a line-pattern of repeat-spacing L_{lines}, the width of the PMMA regions must be $w_{\mathrm{lines}} = L_{\mathrm{lines}} \times f_{\mathrm{Mb}}$.

Dots: For a dot-pattern where the center-to-center distance of cylinders is d_{dots}, the distance between subsequent rows of dots is $L_{\mathrm{dots}} = d_{\mathrm{dots}} \times \sqrt{3}/2$. The radius of each PMMA dot is related to the volume fraction:

$$f_{\mathrm{Mb}} = \frac{\pi r_{\mathrm{dots}}^2}{L_{\mathrm{dots}}^2 (2/\sqrt{3})} \tag{2}$$

$$r_{\mathrm{dots}} = \sqrt{\frac{2 f_{\mathrm{Mb}} L_{\mathrm{dots}}^2}{\sqrt{3}\pi}} \tag{3}$$

Chemical stripe pattern

Consider a simple chemical line-pattern, with repeat-spacing (full period) given by L_{pat}. The chemical pattern consists of alternating stripes of oxide (which attracts PMMA) of width w_{pat}, and stripes of polystyrene brush (which of course attracts PS) of width $L_{\mathrm{pat}} - w_{\mathrm{pat}}$; the duty-cycle is thus $w_{\mathrm{pat}}/L_{\mathrm{pat}}$.

BCP on chemical stripe pattern

When a BCP material orders on a chemical pattern, the morphology is strongly influenced by the underlying pattern. The driving force for this is the same chemical incompatibility that drives the two materials of the BCP to phase-separate and form a nanoscale morphology: the immiscibility given by χ. The BCP material will organize itself to maximize favorable interactions (PMMA on top of oxide; PS on polystyrene brush) and minimize the less-favorable cross-interactions (PMMA

on polystyrene; PS on oxide). A pure BCP can attempt to accommodate an underlying pattern in a few ways: reorienting the morphology, distorting the morphology (stretching or compressing the equilibrium repeat-spacing), or converting to an entirely different morphology. This last option is generally difficult for BCPs, since the block-fraction (f) will define a single energetically-preferred morphology (the one that minimizes interfacial area between the blocks). A blend of BCP materials is demonstrably more responsive in this regard: it is able to adopt either of the parent morphologies, since either morphology requires some energy compromise (stretching/compression of BCP chains). Experimentally, we have established that a $\phi \approx 0.5$ blend forms, on a neutral substrate, a mixture of dots and lines; that is, there is a very small energy difference between these two morphologies.

When a given morphology (dots or lines) assembles on a chemical pattern, the intrinsic BCP ordering will necessarily distort to maximize overlap with the chemical pattern. A BCP with equilibrium repeat-spacing L_0 will distort until its repeat-spacing L matches the underlying pattern L_{pat}. This distortion, of course, involves chain compression/stretching. We can similarly analyze the BCP blend by treating it as a system having an equilibrium L_0 defined by the blending ratio.

Chain stretching

In a BCP blend being ordered by a chemical pattern, the BCP chains will not be in their equilibrium configuration. In particular, the chains will need to compress or stretch in order to accommodate the non-native periodicity (L) imposed by the chemical pattern. Consider a Gaussian chain of N repeat units (each of length a). The radius of gyration is:

$$R_g^2 = \frac{a^2 N}{6} \tag{4}$$

$$R_g = \frac{a N^{1/2}}{6^{1/2}} \tag{5}$$

The stretching of such a chain to a distance R follows:[1]

$$F_{\text{stretch}} = k_B T \frac{3R^2}{2a^2 N} \tag{6}$$

$$\beta F_{\text{stretch}} = \frac{1}{4}\left(\frac{R}{R_g}\right)^2 \tag{7}$$

Where we have defined $\beta = 1/k_B T$. Adding in various realistic conditions (self-avoidance, solvent effects) will modify the pre-factor and the exponent. E.g. for a real chain in a good solvent in 3D, the prefactor is 1.084 and the exponent 2.42.[1] Nevertheless, using $\beta F \approx (R/R_g)^2$ is a reasonable approximation in the general case. Conversely, the compression of a chain induces an energy penalty for confinement of:

$$\beta F_{\text{confine}} = \frac{\pi^2 a^2 N}{6R^2} = \pi^2 \left(\frac{R_g}{R}\right)^2 \tag{8}$$

This form is expected for 'weak confinement' ($R/R_g \approx 1$);[1,2] 'strong confinement' ($R/R_g \ll 1$) would modify the exponent to $\sim 2/3$.[3–6] As expected (Supplementary Figure 5, red line), the potential exhibits roughly spring-like behavior near R_g. Combining the results (c is a constant):

$$\beta F_{\text{chain}} = \begin{cases} c\left(\frac{R}{R_g}\right)^{-2} & \text{if } R < R_g \\ c\left(\frac{R}{R_g}\right)^{2} & \text{if } R > R_g \end{cases} \tag{9}$$

Within a bulk morphology, block-copolymer chains are not, however, found at their unperturbed R_g. This is because of the constraints of the morphology. Phase-separation includes a driving force to minimize the interfacial area (due to the chemical-mismatch, χ). Minimizing the interfacial area can be accomplished by stretching the BCP chains (thereby reducing the average area per chain). A compromise is reached, counter-balancing chain-distortion and interfacial energies, whereupon chains are stretched to R_{eq}. Experimentally, this chain-stretching is observed to be on the order of $R_{\mathrm{eq}}/R_g \approx 1.2$ to 2.0.[7,8] As a rough guide, we thus include a $F_\chi \sim 1/R$ energetic contribution, which accounts for interfacial effects (and, implicitly, other driving forces in BCP ordering). This contribution (Supplementary Figure 5, blue line) shifts the minimum in the total energy per chain (F_{total}, black line) to R_{eq}. This allows us to define L_0 as the equilibrium repeat-spacing in the BCP morphology. Any distortion of the morphology involves a corresponding distortion to the polymer chains.

Distortion of BCP morphology

We define $\epsilon = L_{\mathrm{pat}}/L_0$ as the distortion-factor for the BCP unit-cell. That is, when the BCP morphology assembles on a chemical stripe-pattern of spacing L_{pat}, the unit-cell will expand/contract accordingly to match. Owing to volume conservation, any contraction of the unit-cell along one direction will result in a corresponding expansion along the orthogonal direction. For instance, compressing a line-pattern results in thinner lines (with corresponding expansion along the line long-axis), while compressing hexagonal dot-patterns results in ellipsoidal dots on a distorted hexagonal lattice. Supplementary Figure 6 emphasizes how distorting a BCP unit-cell leads to a corresponding distortion of the BCP chains. The chain distortion of course depends on the angle of the chain within the unit cell:

$$\frac{L}{L_0} = \left(\epsilon^2 \cos^2\theta + \frac{\sin^2\theta}{\epsilon^2} \right)^{1/2} \tag{10}$$

This immediately points to a key difference between line-patterns and dot-patterns: in line-patterns (BCP lamellar phases), the polymer chains are all oriented orthogonal to the lines (i.e. along the repeat-spacing). We define this as $\theta = 0°$ (of course, there are also chains at $\theta = \pm 180°$). In a dot-pattern (BCP hexagonal cylinder phase), the polymer chains are again orthogonal to the domain interfaces, but the local cylindrical symmetry means that chains are oriented at all possible angles ($-180°$ to $+180°$). This modifies the response of the morphology to distortion. Specifically, we expect the total energy cost for the unit-cell to be:

$$\beta F_{\mathrm{u.c.}} = \int_{\theta=-180°}^{+180°} f(\theta) F_{\mathrm{chain}}(L(\epsilon,\theta)) \mathrm{d}\theta \tag{11}$$

where $f(\theta)$ is the orientation-distribution for the chains in the given morphology. I.e. for lines $f(\theta)$ is a narrow peak (Gaussian or delta-function) near $\theta = 0°$, whereas for dots we expect a uniform distribution across all θ. (Note that we are ignoring that in an actual hexagonal morphology, chains at different angles have slightly different amounts of chain-extension, in order to space-fill the entire morphology.) The energy-penalties for chains at different orientations are shown in Supplementary Figure 7. The combined effect for the entire unit-cell (based on Equation 11) is shown in Supplementary Figure 8.

As depicted in Supplementary Figure 8, the energies of the line and dot-patterns are equal for $\epsilon = 1$, consistent with the experimental observation of coexisting morphologies on neutral substrates. This further implies that dot-patterns are lower-energy for all ϵ. However, on a chemically-patterned substrate, there may be an energetic preference for one morphology vs. the other,

depending on their relative overlap with the chemical pattern. Thus, we can imagine that the two energy curves are shifted. This is depicted in Supplementary Figure 8 (right), where different amounts of ϵ lead to different morphologies.

Lines on chemical stripe pattern

If a BCP line phase forms on top of a line-pattern, there will be a strong driving-force for the BCP repeat-spacing to be matched to the chemical pattern ($L_{\text{lines}} = L_{\text{pat}}$). However, because the BCP line-width is dictated by the volume-fraction (f), it is not guaranteed that this width will match the pattern ($w_{\text{lines}} \neq w_{\text{pat}}$). As a result, there will be some surface area of energetic mismatch. In particular, the *relative* mismatched area will follow:

$$A_m = \frac{|w_{\text{pat}} - L_{\text{pat}} \times f_{\text{M}}|}{L_{\text{pat}}} \tag{12}$$

$$= \left| \frac{w_{\text{pat}}}{L_{\text{pat}}} - f_{\text{M}} \right| \tag{13}$$

This is depicted in Supplementary Figure 9. The free-energy cost associated with such a configuration will scale with this mismatch-area, A_m (multiplied by χ).

Dots on chemical stripe pattern

Similarly, when a BCP dot phase forms on top of a line-pattern, there will be a driving force for the MMA cylinder cores to overlap (as much as possible) with the oxide stripes. This will involve a distortion of the morphology so that the rows of dots overlap with the underlying lines ($L_{\text{dots}} = L_{\text{pat}}$). However, there will be an inevitable mismatched area between the MMA cylinder cores and the chemical pattern. When the dots are smaller than the chemical stripes ($2r_{\text{dots}} < w_{\text{pat}}$), we expect:

$$A_m = \frac{w_{\text{pat}} \times d_{\text{dots}} - \pi r_{\text{dots}}^2}{d_{\text{dots}} \times L_{\text{pat}}} \tag{14}$$

$$= \frac{w_{\text{pat}}}{L_{\text{pat}}} - f_{\text{M}} \tag{15}$$

If the dots are larger than the chemical stripes ($2r_{\text{dots}} > w_{\text{pat}}$), the mismatched area is more complex:

$$A_m = \frac{2r_{\text{dots}}^2}{d_{\text{dots}} L_{\text{pat}}} \left(2 \arccos\left(\frac{w_{\text{pat}}}{2r_{\text{dots}}} \right) - \frac{\pi}{2} \right) + \frac{w_{\text{pat}}}{L_{\text{pat}}} - \frac{2 w_{\text{pat}} r_{\text{dots}}}{d_{\text{dots}} L_{\text{pat}}} \sqrt{1 - \left(\frac{w_{\text{pat}}}{2r_{\text{dots}}} \right)^2} \tag{16}$$

The mismatch area for line and dots patterns is compared in Supplementary Figure 9.

Phase diagram

We can combine the above results to predict the expected response of a BCP blend across a range of conditions. The corresponding phase diagram is shown in Supplementary Figure 10, where the horizontal axis is the pitch of the underlying chemical pattern, the vertical axis is the duty cycle of the pattern (i.e. the ratio of the stripe width to the repeat distance), and the color-scale denotes the relative energies of the dot and line-patterns. In the central region of the diagram (purple

lobe), lines are lower-energy; thus when the pitch of the chemical pattern is close to the repeat-spacing of the blend, line-patterns are preferred. In the outer regions (green areas), substantial chain distortion causes dots to instead be lower-energy, and thus preferred. In the transition regions (white), one would expect to see mixtures of dots and lines. Note that this simple model is only applicable for modest distortions of the unit cell ($\epsilon \sim 1$). More complex phenomena are likely to arise (new morphologies, BCP no longer tracking chemical pattern, etc.) under extreme distortions. Thus, the predicted reappearance of lines for low ϵ may not be physically-meaningful.

This theoretical phase diagram is consistent with the experimental data. The data presented in the main text (at a given dose) are horizontal cuts through this diagram. At fixed electron-beam dose, the duty cycle is not strictly conserved; for simplicity, consider traces through the phase diagram that go as $w_{\mathrm{pat}}/L_{\mathrm{pat}} \approx e^{-\epsilon/\mathrm{dose}}$, and that the fraction of lines (f_{l}) and dots (f_{d}) are exponential in the energy difference ($\Delta F = F_{\mathrm{dots}} - F_{\mathrm{lines}}$):

$$f_{\mathrm{l}} = \frac{1}{e^{-\beta \Delta F} + 1} \tag{17}$$

The predicted area fraction for lines (Supplementary Figure 11) exhibits the experimentally-observed features: a plateau where lines are preferred, falling sharply into dot-patterns for smaller pitches, and decaying more slowly into dot-patterns for larger pitches. The dose dependence is also recovered, with mixed morphologies dominating for non-optimal doses. This also highlights that one can select between line and dot-patterns in two complementary ways: by changing the pitch (at fixed dose or duty cycle), or by changing the dose (at fixed pitch).

Additional considerations

We have neglected the fact that the BCP morphology may rearrange in three-dimensions (3D). E.g., different BCP chains (cylinder vs. lamellae) could migrate vertically, in order to enrich the PMMA volume fraction at the substrate, to better-match the underlying chemical pattern. Experimentally, using cross-sectional SEM analysis, we do not observe any strong evidence for such 3D structuring. I.e. the line-patterns look like lamellae, while the dots look like conventional cylinders. Nevertheless, it is likely that such chain rearrangements do play some role in the real system.

Supplementary References

[1] Skvortsov, A. M.; Klushin, L. I.; Birshtein, T. M. Stretching and compression of a macromolecule under different modes of mechanical manupulations. *Polymer Science Series A* **2009**, *51*, 469–491.

[2] Hsu, H.-P.; Grassberger, P. Polymers confined between two parallel plane walls. *The Journal of Chemical Physics* **2004**, *120*, 2034–2041.

[3] Khokhlov, A. R.; Semenov, A. N. Liquid-crystalline ordering in the solution of long persistent chains. *Physica A: Statistical Mechanics and its Applications* **1981**, *108*, 546–556.

[4] Khokhlov, A. R.; Semenov, A. N. Liquid-crystalline ordering in the solution of partially flexible macromolecules. *Physica A: Statistical Mechanics and its Applications* **1982**, *112*, 605–614.

[5] Hsu, H.-P.; Binder, K. Semi-flexible polymer chains in quasi-one-dimensional confinement: a Monte Carlo study on the square lattice. *Soft Matter* **2013**, *9*, 10512–10521.

[6] Smyda, M. R.; Harvey, S. C. The Entropic Cost of Polymer Confinement. *The Journal of Physical Chemistry B* **2012**, *116*, 10928–10934.

[7] Almdal, K.; Rosedale, J. H.; Bates, F. S.; Wignall, G. D.; Fredrickson, G. H. Gaussian- to stretched-coil transition in block copolymer melts. *Physical Review Letters* **1990**, *65*, 1112–1115.

[8] Antonietti, M.; Heinz, S.; Schmidt, M.; Rosenauer, C. Determination of the Micelle Architecture of Polystyrene/Poly(4-vinylpyridine) Block Copolymers in Dilute Solution. *Macromolecules* **1994**, *27*, 3276–3281.
